# Injuries and Post-Traumatic Stress following Historic Tornados: Alabama, April 2011

**DOI:** 10.1371/journal.pone.0083038

**Published:** 2013-12-18

**Authors:** Thomas Niederkrotenthaler, Erin M. Parker, Fernando Ovalle, Rebecca E. Noe, Jeneita Bell, Likang Xu, Melissa A. Morrison, Caitlin E. Mertzlufft, David E. Sugerman

**Affiliations:** 1 Centers for Disease Control and Prevention (CDC), National Center for Injury Prevention and Control (NCIPC), Division of Unintentional Injury Prevention, Atlanta, Georgia, United States of America; 2 Centers for Disease Control and Prevention (CDC), Scientific Education and Professional Development Program Office, Division of Applied Sciences, Epidemic Intelligence Service, Atlanta, Georgia, United States of America; 3 Current affiliation: Medical University of Vienna, Center for Public Health, Department of General Practice and Family Medicine, Vienna, Austria; 4 Centers for Disease Control and Prevention (CDC), National Center for Environmental Health, Division Of Environmental Hazards & Health Effects, Atlanta, Georgia, United States of America; 5 Centers for Disease Control and Prevention (CDC), National Center for Injury Prevention and Control, Division of Analysis, Research, and Practice Integration, Atlanta, Georgia, United States of America; 6 Centers for Disease Control and Prevention (CDC), Office of Public Health Preparedness and Response, Atlanta, Georgia, United States of America; 7 Alabama Department of Public Health (ADPH), Montgomery, Alabama, United States of America; 8 Agency for Toxic Substances and Disease Registry (ATSDR), Division Of Toxicology And Human Health Sciences, Geospatial Research, Analysis And Services Program, Atlanta, Georgia, United States of America; University of Oxford, United Kingdom

## Abstract

**Objectives:**

We analyzed tornado-related injuries seen at hospitals and risk factors for tornado injury, and screened for post-traumatic stress following a statewide tornado-emergency in Alabama in April 2011.

**Methods:**

We conducted a chart abstraction of 1,398 patients at 39 hospitals, mapped injured cases, and conducted a case-control telephone survey of 98 injured cases along with 200 uninjured controls.

**Results:**

Most (n = 1,111, 79.5%) injuries treated were non-life threatening (Injury Severity Score ≤15). Severe injuries often affected head (72.9%) and chest regions (86.4%). Mobile home residents showed the highest odds of injury (OR, 6.98; 95% CI: 2.10–23.20). No severe injuries occurred in tornado shelters. Within permanent homes, the odds of injury were decreased for basements (OR, 0.13; 95% CI: 0.04–0.40), bathrooms (OR, 0.22; 95% CI: 0.06–0.78), hallways (OR, 0.31; 95% CI: 0.11–0.90) and closets (OR, 0.25; 95% CI: 0.07–0.80). Exposure to warnings via the Internet (aOR, 0.20; 95% CI: 0.09–0.49), television (aOR, 0.45; 95% CI: 0.24–0.83), and sirens (aOR, 0.50; 95% CI: 0.30–0.85) decreased the odds of injury, and residents frequently exposed to tornado sirens had lower odds of injury. The prevalence of PTSD in respondents was 22.1% and screening positive for PTSD symptoms was associated with tornado-related loss events.

**Conclusions:**

Primary prevention, particularly improved shelter access, and media warnings, seem essential to prevent severe tornado-injury. Small rooms such as bathrooms may provide some protection within permanent homes when no underground shelter is available.

## Introduction

On April 27, 2011, the third deadliest tornado outbreak in recorded U.S. history hit several southeastern states, with the five most violent Enhanced Fujita (EF) [1] Scale 4 or 5 tornados occurring in Alabama between 2 and 7 p.m.[2], [3] The event resulted in a rare statewide emergency, with 46 hospitals predominantly located in central and northern Alabama reporting a substantial surge of patients. The Alabama Department of Public Health (ADPH) requested epidemiologic assistance from the Centers for Disease Control and Prevention (CDC) to evaluate patterns and risk factors for tornado-related injuries and determine the mental health impact following the disaster.

Known risk factors for injury during tornados include vulnerable home construction,[4], [5] and lack of underground shelter options, such as basements and storm shelters.[6] People outside of structures or in mobile homes during a tornado are at the highest risk of death.[7], [8] If underground shelter is unavailable, closets and bathrooms have been recommended in preparedness guidelines.[4], [9], [10] Empirical research on the effects of warning messages preceding tornado impact is scarce. It has been discussed that sirens and media warnings may improve sheltering behavior,[11]–[14] but frequent exposure to false alarms may conversely lead to desensitization and reduced shelter-seeking.[3], [14]

The objectives of the present study were to characterize tornado injuries treated between April 27–30, 2011 at Alabama hospitals based on a hospital chart review, and to conduct follow-up telephone interviews to identify risk factors and screen for PTSD symptoms.

## Materials and Methods

### Hospital chart abstraction

According to ADPH, 46 hospital emergency departments (EDs) reported patients with tornado-related injuries. Thirty-nine hospitals, including two level I and one level II trauma centers participated. The hospitals were requested to pre-screen their files for all adult (≥18 years) patient charts with ≥1 injury based on International Classification of Diseases, 9th edition, Clinical Modification (ICD-9-CM) codes 800.0-959.9 treated between April 27–30, 2011.This process yielded 2,812 patient charts. Trained CDC and ADPH staff then screened these charts between December 5, 2011 and January 30, 2012. To qualify as a tornado-related injury, in addition to an ICD-9-CM injury code, a time of presentation consistent with tornado impact was required without an alternative explanation for injury. From the screened charts, duplicates (n = 22) and charts unrelated to tornado-injury (n = 1,094) were excluded. Pediatric injuries [15] (ages <18 years old; n = 298) were excluded due to Institutional Review Board (IRB) considerations. We abstracted 1,398 charts.

Abstracted data included demographic information, location at the time of injury, visit type (ED visit or hospitalization), mode of transport to the hospital, discharge disposition, and injury type (direct or indirect injuries). *Direct injuries* included those by flying debris and structural collapse, while *indirect injuries* occurred after the storm due to conditions created by the storm. Abstracted data were entered online using SurveyMonkey (www.surveymonkey.com). We calculated body-region specific Abbreviated Injury Scale (AIS) scores and Injury Severity Scores (ISS) using ICDMAP-90 software,[16] which converts ICD-9-CM codes to AIS and ISS. An ISS of <10 was minor, 10–15 was moderate, and >15 was severe.[17]

### Telephone case-control study

Trained volunteers from CDC and local universities (n = 98) conducted scripted interviews between January 17–31, 2012 at CDC's Emergency Operations Center. A case was an adult person who sustained a tornado-related injury requiring ED care. Controls were uninjured persons who were either in the tornado path or actively avoided being in the path. For case recruitment, we requested patient contact information from the 39 hospitals visited during chart abstraction. Eighteen hospitals provided patient phone numbers, while 4 mailed letters requesting that cases contact ADPH. Eleven hospitals declined. We received contact information for 419 abstracted patients. One hundred eighty-five (44.2%) had a disconnected number, 52 (12.4%) had an incorrect number, 20 (4.8%) were duplicates, and 57 (20.0%) declined participation. Of 105 (25.1%) who started the interview, 98 (23.4%) completed, yielding a response rate of 60.5%.[18]

Controls were recruited with public service announcements aired in English from January 3–10, 2012 in the TV markets affected. Respondents left contact information on the ADPH website (n = 173) or by a toll-free automated telephone mailbox (n = 84). An additional 97 controls were referred by interviewed individuals. The eligibility of respondents was self-screened prior to leaving contact information, and eligibility was verified during the interviews. Six (1.7%) had a disconnected number, 5 (1.4%) had an incorrect number, 13 (3.7%) were duplicates, and 121 (34.2%) declined participation. Of 209 (59.0%) who started the interview, 200 (56.5%) completed the survey, yielding a response rate of 60.6%.[18] The pretested survey included address and type of location at the time of tornado impact, room type, presence of windows, floor level, and structure of material and foundation. We also asked if helmets were used for head protection, which warning sources (e.g., TV, or ‘word of mouth’ community warnings) persons had access to and which sources were actually used. We asked about the reaction to the sirens, the estimated annual frequency of tornado siren exposure, and past experiences with tornados being seen after hearing sirens.

We screened all surveyed individuals for PTSD symptoms using Breslau's Short Screening Scale.[19] This tool has seven dichotomous questions and a cut-off value of four points has demonstrated a sensitivity of 80% and a specificity of 97% in detecting PTSD.[19] Several variables that may increase the risk of PTSD were assessed during the interviews using questions from the Behavioral Risk Factor Surveillance System.[20] These variables included loss events (loss of a close person or pet, the home, or the workplace due to destruction caused by the storm), a psychiatric history prior to the tornado, and sustaining a tornado-related injury. Referrals to Project Rebound (http://www.projectrebound.ua.edu/), which has provided counseling to tornado victims were offered to all respondents at the end of the interviews. Surveyed data were entered online using SurveyMonkey (www.surveymonkey.com).

### Geocoding

We geocoded the physical address at the time of tornado impact using ESRI ArcMap 10.0 (www.esri.com) for all chart-abstracted and interviewed cases and controls with a known address (total N = 467). We created a composite of the tornado tracks from data available from the National Weather Service (NWS) post-storm damage assessments [3], [21] to calculate proximity of cases and controls to tornado tracks. This approach was taken to control for potential differences in tornado exposure for cases and controls in the subsequent analysis of associations between injury and location during the time of tornado impact.

### Statistical analysis

#### Chart abstraction data

Frequency distributions were computed for all variables abstracted from medical charts. Associations of abstracted variables with injury severity were evaluated using mean score chi-square tests when the outcome variable was ordinal and the independent variable was categorical. Correlation chi-square tests were used to calculate associations of ordinal dependent with ordinal independent variables. For cells with sample size <5, Fisher's exact test was used. Further, we obtained census block data on sex and age to analyze differences between surveyed individuals and the total population in the area.

#### Survey data on respondent's location at the time of tornado impact

We used the frequency procedure (Proc Freq) in SAS with an odds ratio (OR) option to calculate crude odds ratios for injury associated with respondent's location at the time of tornado impact. For cells with small sample size (<5), exact tests were used to calculate odds ratios. Adjusted associations between the respondent's location at the time of tornado impact with injury were assessed with conditional logistic regression and adjusted for the respondent's distance from the closest tornado path at the time of tornado impact, age, sex and race. We adjusted these analyses for the respondent's distance to the closest tornado track in order to control for differences in actual exposure to the tornado between cases and controls, which would potentially bias risk estimates for different types of structures and locations during the storm.

#### Survey data on warning systems and reactions shown to sirens

In order to calculate the odds of injury for (a) exposure to different warning systems, (b) annual estimated frequency of siren exposure, (c) past tornado exposure after hearing sirens and (c) specific types of reactions to sirens shown during the event, we used the frequency procedure (Proc Freq) in SAS with an odds ratio (OR) option to calculate odds ratios. For multivariate analyses, we used conditional logistic regression analyses. Exact versions of the tests were applied for analyses of variables with small cell (<5).

Control variables for all analyses performed were carefully selected based on theoretical reasoning. Because of partially small sample sizes, only a limited number of control variables could be selected. Warning systems were adjusted for age, sex, race and access to the respective warning system. This approach was taken because media use (e.g., internet use) has been shown to vary widely between demographic populations, e.g. across age groups.[22] Access to the respective warning system was included as a control variable in these analyses in order to control for differences in the availability of each system across households and regions.

The annual estimated frequency of siren exposure as an explanatory variable for injury was similarly adjusted for age, sex and race and additionally adjusted for past experiences with witnessing a tornado after hearing past tornado sirens. The latter variable was included here because witnessing no tornado after hearing tornado sirens in the past may desensitize individuals and reduce the effect of tornado siren exposure on injury-protective behaviors.[14] Following a similar adjustment scheme, past experiences with tornado exposure after hearing sirens as a protective factor was adjusted for age, sex, race, and the frequency of annual siren exposure. Specific types of reactions to sirens shown on April 27, 2011 were controlled for the respondent's age, sex, and race because reactions may vary with demographic characteristics.

#### Survey data on PTSD

In the analysis of risk factors for PTSD, positive screening for PTSD (across all surveyed individuals) was the outcome variable. We used the frequency procedure (Proc Freq) in SAS with an odds ratio (OR) option to calculate crude odds ratios. For multivariate analyses, we used conditional logistic regression analyses. The odds ratios of PTSD for specific types of loss events and number of loss events were all adjusted for the respondent's age, sex and race because of known differences in the risk for PTSD across demographic groups.[23] Additionally, we controlled these analyses for the respondent's injury sustained during the tornado (which may reflect a higher degree of traumatization and/or exposure to the storm), and for the respondent's psychiatric history before the tornado emergency. The latter variable was included because previous psychiatric morbidity increases the risk of traumatization and PTSD.[23] Psychiatric history prior to the tornado as a risk factors for PTSD was similarly adjusted for age, sex, and race, the number of sustained loss events sustained during the tornado, and own tornado injury. Following the same adjustment scheme, own tornado injury as risk factor for PTSD was adjusted for age, sex, race, number of loss events and past psychiatric history.

Across all analyses of survey data, missing values were coded as a separate category. We used SAS 9.3 for Windows for all statistical analysis.

#### Ethics Statement

Ethical approval was obtained from U.S. Department of Health and Human Services/Centers for Disease Control and Prevention (CDC) Institutional Review Board (Atlanta, Georgia) and from the Alabama Department of Public Health Institutional Review Board (Montgomery, Alabama). Minimal data use agreements were signed with all 39 participating hospitals. These hospital were (in alphabetical order): Athens Limestone Hospital, Bibb Medical Center, Brookwood Medical Center, Bryan W. Whitfield Memorial Hospital, Cherokee Medical Center, Community Hospital Tallassee, Crestwood Medical Center, Cullman Regional Medical Center, DCH Health System Northport Medical Center, DCH Regional Medical Center Tuscaloosa, Decatur Morgan Hospital, DeKalb Regional Medical Center, East Alabama Medical Center, Eliza Coffee Memorial Hospital, Gadsden Regional Medical Center, Hale County Hospital, Hartselle Medical Center, Helen Keller Hospital, Highlands Medical Center, Hill Hospital Of Sumter County, Huntsville Hospital, Jacksonville Medical Center, Lawrence Medical Center, Marshall Medical Center North, Marshall Medical Center South, Northwest Medical Center, Parkway Medical Center, Pickens County Medical Center, Princeton Baptist Medical Center, Red Bay Hospital, Russell Medical Center, Russellville Hospital, Shoals Hospital, St. Vincent's Birmingham, St. Vincent's Blount, St. Vincent's East, UAB Hospital Birmingham, UAB Medical West, Walker Baptist Medical Center. All surveyed individuals gave their oral consent to the survey at the beginning of the interviews. Documentation of verbal consent was obtained within the online survey tool SurveyMonkey (www.surveymonkey.com) for all respondents. Verbal consent was obtained as opposed to written because respondents were interviewed from a call center in Atlanta across multiple locales in Alabama. Public Health investigations like this investigation, which follow the ‘Epi-Aid’ mechanism designed to provide federal epidemiological assistance from CDC to states typically don't require written consent. The consent procedure was approved by the Institutional Review Boards.

## Results

As indicated by data from chart abstraction, most of the tornado-related injuries with known ISS (n = 1,170) were relatively minor (n = 1,041, 89%), though 6% (n = 70) were moderate, and 5% (n = 59) were severe (Table 1). More than 70% were directly related to tornado impact, while indirect injuries were predominantly from clean-up activities, such as improper use of chainsaws and other powertools (n = 40, 14%), puncture wounds (n = 43, 15.2%), or from falling objects (n = 55, 20%) (data not shown). Those with direct injuries were more severely injured than patients with indirect injuries, _X_^2^ (1, N = 1,100) = 21.13, p<.0001 (Table 1). Less than a quarter of all injured patients were admitted, with a length of stay from 1 to 92 days. Of those admitted, more than 20% were sent to an intensive care unit (ICU) for 1 to 30 days (data not shown). Most patients were discharged home (n = 1,202, 86%), while 15 (1%) died prior to discharge (Table 1).

**Table 1:** Characteristics of patients with tornado injuries seen in emergency departments based on chart review—Alabama, April 27–30, 2011.

	Total Injured	Minor	Moderate	Severe	
Characteristics	(N = 1,398)	(ISS^a^ 1–9) N = 1,041	(ISS^a^ 10–15) N = 70	(ISS^a^ >15) N = 59	P-value^f^
	n (%)	n (%)	n (%)	n (%)	
**Sex**					0.01
Female	662 (47.4)	507 (48.7)	32 (45.7)	19 (32.2)	
Male	684 (48.9)	510 (49.0)	38 (54.3)	40 (67.8)	
Unknown	52 (3.7)	24 (2.3)	0 (0)	0 (0)	
**Age Group**					0.05^g^
18–29	257 (18.4)	204 (19.6)	9 (12.9)	11 (18.6)	0.29
30–44	339 (24.2)	264 (25.4)	12 (17.1)	12 (20.3)	0.07
45–64	455 (32.5)	338 (32.5)	28 (40.0)	27 (45.8)	0.05
≥65	230 (16.5)	160 (15.4)	21 (30.0)	8 (13.6)	0.43
Unknown	117 (8.4)	75 (7.2)	0 (0)	1 (1.7)	
**Health Insurance** b					0.88
Private insurance	412 (29.5)	324 (31.1)	22 (31.4)	21 (35.6)	0.62
Medicare	220 (15.7)	160 (15.4)	16 (22.9)	6 (10.2)	0.75
Medicaid	142 (10.2)	100 (9.6)	9 (12.9)	3 (5.1)	0.49
Self pay	472 (33.8)	372 (35.7)	20 (28.6)	25 (42.4)	0.84
Unknown	152 (10.9)	85 (8.2)	3 (4.3)	4 (6.8)	
**Injury Location**					<0.001
Apartment	23 (1.6)	17 (1.6)	0 (0)	1 (1.7)	0.65
House	326 (23.3)	239 (23.0)	25 (35.7)	8 (13.6)	0.62
Mobile home	105 (7.5)	77 (7.4)	9 (12.9)	11 (18.6)	<0.001
Unspecified home	184 (13.2)	136 (13.1)	9 (12.9)	10 (16.9)	0.48
Motor vehicle	56 (4.0)	41 (3.9)	3 (4.3)	3 (5.1)	0.67
Outdoors	170 (12.2)	150 (14.4)	4 (5.7)	2 (3.4)	0.001
Public or commercial building	34 (2.4)	26 (2.5)	3 (4.3)	2 (3.4)	0.45
Unspecified storm shelter^c^	8 (0.6)	6 (0.6)	0 (0)	0 (0)	0.41
Unknown	492 (35.2)	349 (33.5)	17 (24.3)	22 (37.3)	
**Direct vs. Indirect Injury** d					<0.0001
Direct injury	1,023 (73.2)	753 (72.3)	63 (90.0)	53 (89.8)	
Indirect Injury	282 (20.2)	225 (21.6)	5 (7.1)	1 (1.7)	
Unknown	93 (6.7)	63 (6.1)	2 (2.9)	5 (8.5)	
**Prehospital Transport**					<0.0001
Private Vehicle	534 (38.2)	449 (43.1)	7 (10.0)	2 (3.4)	<0.0001
Walk-in	21 (1.5)	19 (1.8)	1 (1.4)	1 (1.7)	0.78
Ground ambulance	435 (31.1)	296 (28.4)	44 (62.9)	43 (72.9)	<0.0001
Helicopter ambulance	7 (0.5)	3 (0.3)	1 (1.4)	3 (5.1)	<0.0001
Unknown	401 (28.7)	274 (26.3)	17 (24.3)	10 (16.9)	
**Transferred From Another Hospital** e	78 (5.6)	39 (3.7)	14 (20.0)	21 (35.6)	<0.0001
**Injured Body Region** b					<0.0001
Extremities or pelvic girdle	898 (64.2)	764 (73.4)	55 (78.6)	37 (62.7)	<0.0001
Head/face/neck	524 (37.5)	402 (38.6)	38 (54.3)	43 (72.9)	0.56
Chest	326 (23.3)	196 (18.8)	47 (67.1)	51 (86.4)	<0.0001
Abdomen and pelvic contents	238 (17.0)	166 (15.9)	24 (34.3)	39 (66.1)	<0.0001
Disposition					<0.0001
Home/self care	1,202 (86.0)	960 (92.2)	33 (47.1)	19 (32.2)	<0.0001
Rehab/care facility	90 (6.4)	36 (3.5)	24 (34.3)	23 (39.0)	<0.0001
Interfacility transfer	65 (4.6)	36 (3.5)	11 (15.7)	10 (16.9)	<0.0001
Died	15 (1.1)	1 (0.1)	1 (1.4)	6 (10.2)	<0.0001
Unknown	26 (1.9)	8 (0.8)	1 (1.4)	1 (1.7)	

%) of patients.^a^ The ISS was known for 1,170 (83.7

^b^ Patients may be counted more than once due to multiple insurance types or diagnoses.

^c^ Unknown if FEMA approved tornado storm shelter or safe room or a basement in a private residence.

–30.^d^ Direct injuries include those caused by flying debris and structural collapse from the tornado on April 27; indirect injuries occurred nearly exclusively from April 28

^e^ Based on transfer information available in hospital records as well as linking patient records between hospitals. Patients with missing unique identifiers (name or date of birth) could not be linked.

^f^ Mean score chi-square test or Fisher's exact chi-square test (when sample size <5) used for overall group and each level compared to all other levels combined, unless stated otherwise.

^g^ Based on correlation chi-square test

More patients arrived at hospitals by private vehicles than by ground or helicopter ambulance combined (38% vs. 32%), but individuals transported by ground ambulance were more severely injured as compared to all other patients, _X_^2^ (1, N = 879) = 73.73, p<.0001 (Table 1). The extremities and pelvic girdle (64.2%) were the most frequently injured body regions overall, followed by head injuries (37.5%). Head, chest and abdomen regions were affected in the majority of severe trauma. Head injuries resulted in many hospitalizations (46.5%), most ICU admissions (56.3%), and deaths (71.4%) (data not shown).

Regarding demographic patient variables, males had significantly more severe injuries than females, _X_^2^ (1, N = 1,146) = 6.74, p = .009, and injury severity increased with rising age, although this finding was only borderline significant, _X_^2^ (1, N = 1,094) = 3.76, p = .052 (Table 1). Those aged 30–64 were more frequently injured than those 18–29 or over 65 years, and a slight majority was self-pay. While more injuries occurred in permanent homes (houses and apartments) than in mobile homes (24.9.% of vs. 7.5%, Table 1), individuals in mobile homes were more severely injured than other patients, _X_^2^ (1, N = 1,170) = 11.32, p = .0008 (Table 1).

A comparison of census block data with abstracted data indicated no significant differences between abstracted individuals and adult residents regarding age and sex (data not shown).

A comparison of the demographic characteristics age, sex and race of individuals whose data were abstracted from hospital charts with those who participated in the telephone survey is shown in Table 2. As indicated by chi-square tests, individuals who participated in the survey were more often between 45 and 64 years of age, less frequently male, and less frequently of black or African-American origin as compared to abstracted patients (Table 2). Surveyed cases were, however, not significantly different from surveyed controls in terms of age group (_X_^2^ (4, N = 298) = 2.34, p = .67), sex (_X_^2^ (2, N = 298) = 1.10, p = .58), and race (_X_^2^ (2, N = 298) = 4.94, p = 0.08) (data not shown).

**Table 2:** Demographic characteristics of patients whose hospital charts were abstracted, and of survey participants.

	Data from survey (N = 298) n (%)	Data from chart abstraction (N = 1398) n (%)	Overall P-value^a^	Odds Ratio (95% CI)
**Age Group**			0.005	
18–29	41 (13.8)	257 (18.4)		1 (Reference)
30–44	70 (23.5)	339 (24.2)		1.29 (0.85–1.97)
45–64	135 (45.3)	455 (32.5)		1.86 (1.27–2.72)
65+	46 (15.4)	230 (16.5)		1.25 (0.79–1.98)
Unknown	6 (2.0)	117 (8.4)		
**Sex**			<.0001	
Female	193 (64.8)	662 (47.4)		1 (Reference)
Male	103 (34.6)	684 (48.9)		0.52 (0.40–0.67)
Unknown	2 (0.7)	52 (3.7)		
**Race**			<.0001	
White	243 (81.5)	862 (61.7)		1 (Reference)
Black or African-American	45 (15.1)	342 (24.5)		0.47 (0.33–0.66)
Other/unknown	10 (3.4)	194 (13.9)		

^a^ Based on chi-square tests.

**Figure 1:**
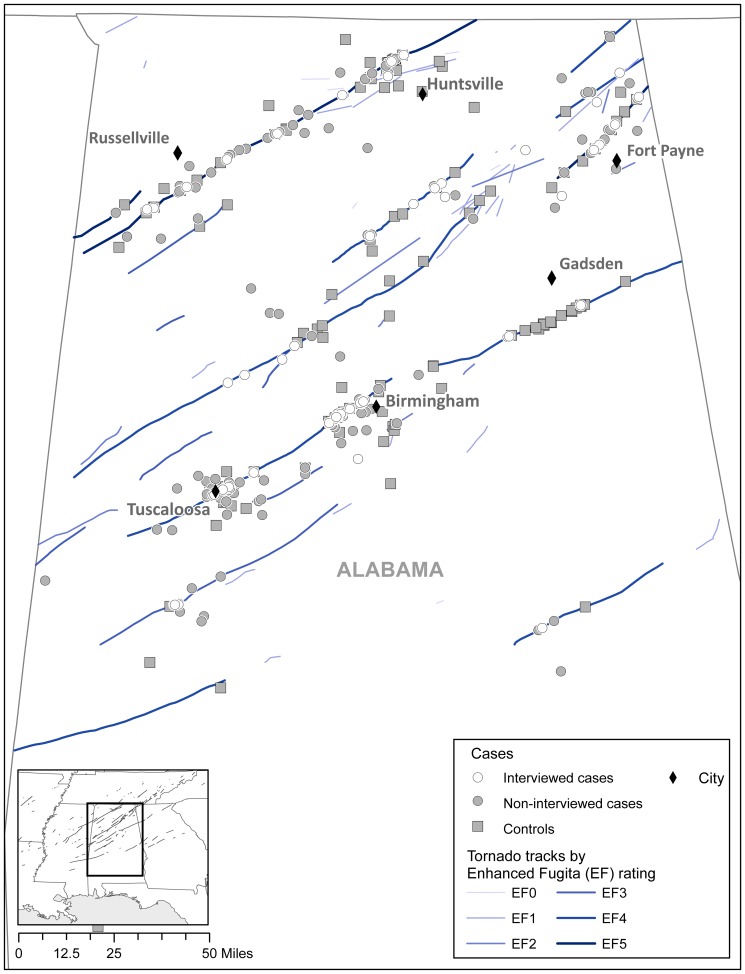
Geographic Information System (GIS) mapping of locations at the time of the tornado for 183 uninjured interviewed controls and 284 injured cases (including 73 interviewed cases and 211 non-interviewed patients with geocodable information in their hospital chart). A layer indicating the tornado paths was added. Most of the mapped cases (83.0%) and controls (83.3%) were closest to a violent EF 4–5 tornado path. EF tornado rating scale estimates the strongest wind gusts that occur 10 meters above the ground: EF-0 (65–85 mph [105–137 km/h]), EF-1 (86–110 mph [138–177 km/h]), EF-2 (111–135 mph [178–217 km/h]), EF-3 (136–165 mph [218–266 km/h]), EF-4 (166–200 mph [267–322 km/h]), and EF-5 (>200 mph [>322 km/h]).[1]

Figure 1 visualizes the geocoded locations of cases and uninjured controls along with registered tornado paths and EF-categories. Controls were further away from tornado tracks than surveyed cases (0.95 miles vs. 0.46 miles, p = 0.0001). Surveyed cases were closer to the nearest tornado track than non-interviewed cases (mean distance in miles 0.46 vs. 1.1, p = .004).

Being located in a mobile home at the time of tornado impact compared to a permanent residence had the greatest odds of injury (odds ratio [OR], 6.98; 95% confidence interval [CI]: 2.10–23.20) (Table 3). Within residences, location in a basement (OR, 0.13; 95% CI, 0.04–0.40), bathroom (OR, 0.22; 95% CI: 0.06–0.78), closet (OR, 0.25; 95% CI, 0.07–0.80) and hallway (OR, 0.31; 95% CI, 0.11–0.90) had significantly lower odds for injury compared to being in a living room, kitchen, or family room. This analysis was controlled for the respondent's distance from the closest tornado path at the time of tornado impact as well as age, sex, and race. No injuries were noted in a public/commercial building or storm shelter or among persons wearing helmets (Table 3).

**Table 3:** Odds of injury associated with respondent's location during the time of tornado impact among surveyed cases (N = 73) and controls (N = 183) with non-missing distance to the closest tornado track.

Characteristics	Case (N = 73) n (%)		Control (N = 183) n (%)	Odds Ratio (95% CI)^a^		Adjusted Odds Ratio (95% CI)^a^
**Location Type**						
Permanent residence	56 (76.7)		135 (73.8)	1 (Reference)	1 (Reference)	
Mobile home	11 (15.1)		6 (3.3)	4.42 (1.56–12.53)	6.98 (2.10–23.20)	
Motor vehicle	5 (6.8)		10 (5.5)	1.21 (0.39–3.69)	1.28 (0.35–4.67)	
Storm shelter	0 (0)		16 (8.7)	N/A	N/A	
Public/Commercial building	0 (0)		10 (5.5)	N/A	N/A	
Unknown	1 (1.4)		6 (3.3)			
**Room Type in Residence** b						
Bed/Family/Kitchen	19 (33.9)		18 (13.3)	1 (Reference)	1 (Reference)	
Basement	9 (16.1)		44 (32.6)	0.19 (0.0 7–0.51)	0.13 (0.04–0.40)	
Bathroom/Tub	5 (8.9)	19 (14.1)		0.25 (0.08–0.81)	0.22 (0.06–0.78)	
Closet	8 (14.3)	19 (14.1)		0.40 (0.14–1.14)	0.25 (0.07–0.80)	
Hallway	12 (21.4)	26 (19.3)		0.44 (0.17–1.12)	0.31 (0.11–0.90)	
Unknown	3 (5.4)	9 (6.7)				
**Window in Room** b						
No	26 (46.4)	71 (52.6)		1 (Reference)	1 (Reference)	
Yes	30 (53.6)	63 (46.7)		1.30 (0.70–2.43)	1.21 (0.62–2.39)	
Unknown	0 (0)	1 (0.7)				
**Floor** b						
1^st^	43 (76.8)	80 (59.3)		1 (Reference)	1 (Reference)	
2nd or higher	4 (7.1)	8 (5.9)		0.93 (0.27–3.27)	1.16 (0.28–4.82)	
Basement	9 (16.1)	47 (34.8)		0.36 (0.16–0.80)	0.32 (0.14–0.74)	
**Structure Material** b						
Brick	23 (41.1)	65 (48.1)		1 (Reference)	1 (Reference)	
Wood	31 (55.4)	65 (48.1)		1.35 (0.71–2.56)	1.49 (0.75–2.99)	
Other^c^	2 (3.6)	5 (3.7)		1.13 (0.21–6.23)	1.66 (0.27–10.19)	
**Structure Fundation** b						
Crawl space	12 (21.4)	27 (20)		1 (Reference)	1 (Reference)	
Blocks/pier and beam	12 (21.4)	24 (17.8)		1.13 (0.43–2.97)	0.97 (0.39–2.42)	
Concrete slabs	24 (42.9)	61 (45.2)		0.89 (0.39–2.03)	0.69 (0.26–1.81)	
Unknown	8 (14.3)	23 (17)				
**Helmet use** d						
No	73 (100)	175 (95.6)		1 (Reference)	1 (Reference)	
Yes	0	8 (4.4)		N/A	N/A	

^a^ Controlled for the distance to the closest tornado track at the time of tornado impact, age, sex and race.

= 56 cases, N = 135 controls).^b^ Calculations based on injuries that occurred in permanent residences (N

“Other” includes stone, stucco, cinder block, and steel.^c^

d Includes firefighter helmet, bicycle helmet, and motorcycle helmet.

After adjustment for accessibility of the specific warning source and demographics, exposure to the NOAA weather radios (adjusted odds ratio [aOR], 0.40; 95% CI: 0.19–0.84), TV (aOR, 0.45; 95% CI, 0.24–0.83), or internet warnings (aOR, 0.20; 95% CI, 0.09–0.49), and sirens (aOR, 0.50; 95% CI, 0.30–0.85) showed decreased odds of injury (Table 4). The past experience of witnessing a tornado after hearing tornado sirens was associated with lower odds of injury (aOR, 0.50; 95% CI, 0.28–0.90). Frequent estimated annual tonado siren exposure (>5 times per year) was associated with slightly lower odds of injury on the day of the event (aOR, 0.41, 95% CI, 0.17–0.99). Among the reactions to sirens shown on the day of the event, seeking further information was associated with lower odds of injury (aOR, 0.41; 95% CI, 0.18–0.93).

**Table 4:** Odds of injury associated with exposure and reaction to warning systems among surveyed cases (N = 98) and controls (N = 200).

Warning characteristics	Case(N = 98) N (%)	Control (N = 200) N (%)	Odds Ratio (95% CI)	Adjusted Odds Ratio (95% CI)^a^
**Warning System** b,c				
AM/FM radio	46 (46.9)	103 (51.5)	0.85 (0.52–1.38)	0.81 (0.47–1.40)
NOAA Weather Radio	17 (17.3)	60 (60.3)	0.51 (0.28–0.94)	0.40 (0.19–0.84)
TV	63 (64.3)	158 (79.0)	0.51 (0.30–0.88)	0.45 (0.24–0.83)
Internet	7 (7.1)	53 (26.5)	0.22 (0.10–0.50)	0.20 (0.09–0.49)
Community	43 (43.9)	112 (56.0)	0.64 (0.39–1.05)	0.71 (0.42–1.17)
Sirens	49 (50.0)	131 (65.5)	0.54 (0.32–0.89)	0.50 (0.30–0.85)
No exposure to any	23 (23.5)	35 (17.5)	1.74 (0.95–3.18)	1.39 (0.69–2.78)
**Frequency of Annual Siren Exposure**				
1–5 times	37 (37.8)	81 (40.5)	0.50 (0.25–0.97)	0.44 (0.19–1.02)
>5 times	26 (26.5)	78 (39.0)	0.36 (0.18–0.74)	0.41 (0.17–0.99)
Never	24 (24.5)	26 (13.0)	1 [Reference]	1 (Reference)
Unknown	11 (11.2)	15 (7.5)		
**Past Tornado Exposure after Hearing Sirens**				
No	53 (54.1)	77 (38.5)	1 [Reference]	1 (Reference)
Yes	30 (30.6)	101 (50.5)	0.43 (0.25–0.74)	0.50 (0.28–0.90)
Unknown	15 (15.3)	22 (11.0)		
**Reaction to Siren on April 27, 2011** b,c,d				
Tried to get to shelter	20 (20.4)	61 (30.5)	0.79 (0.41–1.51)	0.92 (0.46–1.80)
Sought information	37 (37.8)	112 (56.0)	0.43 (0.19–0.94)	0.41 (0.18–0.93)
Got in car to flee	4 (4.1)	26 (13.0)	0.34 (0.08–1.08)	0.28 (0.05–1.01)
No location	23 (23.5)	47 (23.5)	1.45 (0.75–2.80)	1.39 (0.69–2.78)

a Each warning system controlled for access to respective system, age, sex and race; frequency of annual siren exposure adjusted for age, sex, race, and past tornado exposure after hearing sirens; past tornado exposure after hearing sirens adjusted for age, sex, race, and the frequency of annual siren exposure; reaction to siren on April 27, 2011 adjusted for age, sex and race.

b Reference category is answer “no” to respective item.

c Multiple positive responses allowed.

d Calculations restricted to individuals who were exposed to sirens that day (N = 49 cases and N = 131 controls).

Sixty-six (22.1%) participants of the 298 surveyed patients screened positive for PTSD (Table 5). Death of a close person in the tornado (aOR, 2.36; 95% CI, 1.22–4.54), loss of one's home (aOR, 3.48; 95% CI, 1.61–7.52), loss of the workplace (due to destruction caused by the tornado) (aOR, 2.91; 95% CI, 1.05–8.06), and a psychiatric history (aOR, 3.94; 95% CI, 2.01–7.70) were all associated with an increased odds for experiencing PTSD symptoms. The odds of screening positive for PTSD increased with the number of loss events (Table 5).

**Table 5:** Odds of post-traumatic stress syndrome (PTSD) symptoms associated with loss events and past psychiatric history among injured and uninjured surveyed participants eight months after tornado impact (N = 298).

Risk Factors for PTSD^a^			PTSD Case (N = 66) N (%)	PTSD Control (N = 232) N (%)	Odds Ratio (95% CI)	Adjusted Odds Ratio (95% CI)^b^		
**Loss Events** c,d								
Loss of close person			30 (45.5)	50 (21.6)	2.97 (1.67–5.28)	2.36 (1.22–4.54)		
Loss of home			52 (78.8)	131 (56.5)	2.96 (1.53–5.74)	3.48 (1.61–7.52)		
Loss of pet			19 (28.8)	45 (19.4)	1.64 (0.88–3.07)	1.07 (0.51–2.23)		
Loss of workplace			10 (15.2)	12 (5.2)	3.16 (1.30–7.68)	2.91 (1.05–8.06)		
**Number of Loss Events**								
None			9 (13.6)	80 (34.5)	1 (Reference)	1 (Reference)		
1 to 2			42 (63.6)	131 (56.5)	2.85 (1.32–6.17)	3.08 (1.30–7.30)		
3 to 4			15 (22.7)	21 (9.1)	6.35 (2.44–16.52)	4.71 (1.52–14.57)		
**Own Tornado Injury** c			26 (39.4)	72 (31.0)	1.44 (0.82–2.55)	1.14 (0.57–2.26)		
**Past Psychiatric History** c			29 (43.9)	33 (14.2)	4.58 (2.49–8.44)	3. 94 (2.01–7.70)		

a PTSD screening based on Breslau's Short Screening Scale for PTSD (7 dichotomous items); 4 points was the cut-off used for presence of PTSD.

b Loss events, and number of loss events adjusted for age, race, sex, own tornado injury and past psychiatric history; own tornado injury adjusted for age, sex, race, number of loss events and past psychiatric history; past psychiatric history adjusted for age, sex, race, number of loss events, and own tornado injury.

c Reference category is answer “no” to respective item.

d Multiple positive responses allowed.

## Discussion

A large surge of injured patients was seen at local hospitals during the statewide emergency. Similar to historic tornado events, most of these injuries were non-severe, with the extremities being the most frequently injured body region.[24]–[30] Injuries to the head were among the most severe injuries, leading to a majority of ICU admissions and deaths. Helmets reduce head injuries in high impact motorcycle crashes,[31] and may also reduce the severity of head injuries following tornados. However, in the present sample, only eight individuals indicated helmet use, and all of them remained uninjured. Because helmets may prevent some head injuries but cannot protect other body regions than the head, other protective factors than helmet use may have been at play in individuals who had helmets on. Helmet users may have generally acted in more protective ways than non-users, or may have accessed or responded to warnings earlier than non-users, which may have reduced their overall risk of injury. Further research seems warranted to test the effects of helmets on injuries in severe storms.

A prior analysis of vital statistics data uncovered 212 deaths in Alabama,[2] while our analysis of hospital charts showed just 15 deaths, indicating death on impact for the vast majority of patients. High proportions of deaths before transportation to hospitals have also been reported based on Red Cross data,[2] underlining the extraordinary forces involved in this tornado emergency. Severely injured patients who survived until hospitalization were predominantly transported by ambulance, but in total more patients were transported by private vehicles, confirming the important role of private helpers in this tornado event.[3]

In contrast to mobile home residents who have repeatedly been shown to sustain more severe injuries,[6]–[8], [32]–[34] the number of injuries in public commercial buildings and storm shelters was very low, underlining the protective benefit of these structures. However, our survey also indicated that the proportion of uninjured controls located in these types of structures was small. An early assessment following the tornados showed that few people had knowledge of a storm shelter close to their home,[3] and affected areas had an inadequate number of shelters.[35] The present findings thereby underline the need for improved access to and use of safe structures in tornado-prone areas.

The current analysis indicates that, if safe structures are out of reach, individuals located in a permanent home, who made up a large proportion of injuries, can still reduce their injury risk. Underground basements,[6], [32], [34] closets, hallways, and bathrooms seemed safer in the present analysis than other rooms. Current tornado preparedness guidelines recommend seeking shelter in interior rooms when there is no basement or tornado shelter available, but their protective effect had rarely been tested.[4], [9], [10]

It is noteworthy that about a quarter of all injuries may be prevented by increasing safety during clean-up. Enhanced public health messaging regarding the safe use of powertools,[35] and proper head, hand and foot protection may be beneficial during clean-up following future events.[36]

The present assessment also provided some novel findings on the potential roles of warning messages preceding tornado impact. Consistent with an earlier study,[14] seeing televised warnings was associated with a lower injury risk, but also warnings from the NOAA weather radios, and particularly the internet, as well as tornado sirens may reduce the risk of injury. There was no support for desensitization resulting from frequent tornado siren exposure.[3], [37] On the contrary, we identified an inverse association between frequency of siren exposure and injury risk. There were also differences in injury risk estimates depending on the reaction to the siren. Seeking further information, which is the most typical immediate reaction to hearing a siren,[14] was associated with a decreased risk of injury. Fleeing in a motor vehicle also showed a decreased but non-significant association with injury. Some,[4], [38] but not all [7] earlier literature indicates that fleeing a tornado in a motor vehicle may be protective under certain circumstances. However, current tornado preparedness guidelines advise against outrunning a tornado by motor vehicle.[10]

It is known that severe storms pose not only a physical but also a psychological burden on the affected population,[39] with up to 66% of affected individuals requiring crisis counseling after a violent tornado,[40] and a prevalence of PTSD of 2% to 59% later on.[41], [42] In our sample, 22.1% of respondents screened positive for PTSD symptoms. In accordance with the literature, loss events and history of psychiatric disorder were risk factors for PTSD symptoms 8 months post-event.[43], [44] These findings underline the need for long-term psychosocial support in affected communities.[40], [44]–[47]

This analysis has several limitations. Seven of the 46 hospitals that saw patients following the tornado did not participate in the chart abstraction. However, these hospitals were mainly located in the periphery of the tornado tracks, and collectively reported seeing only 57 patients. Further, eleven hospitals declined releasing patient contact information for the case-control survey. Regarding the telephone survey, a random selection of cases and controls was not possible, and only cases and controls with current phone numbers or call forwarding could be contacted. The survey findings are therefore based on a convenience sample and not representative of the total population. Further, differences between surveyed cases and controls may have been more likely to be detected because the case patients were closer to the tornado tracks than the participating controls. Cases and controls were interviewed several months after the tornado, which may have increased the risk of recall bias in the telephone survey, and reluctance in answering sensitive questions is a further likely limitation.[48] Some controls for the survey were referred by other survey respondents, which was deemed necessary to increase the number of participants, but may potentially have introduced selection bias.[48] The number of surveyed cases and controls in some categories (e.g., location in a mobile home at the time of tornado impact) were small, and confidence intervals relatively wide. The comparison of chart abstraction data with data from participants in the telephone survey indicated that males and black/Afro-American individuals were underrepresented in the survey, while those in the age range of 45 to 64 years were overrepresented. Finally, case numbers for several categories were small, resulting in limited statistical power, and cross-sectional studies generally do not allow to specify cause and effect.

## Conclusions

This study replicated some previous findings suggesting a high risk of injury during violent tornados for persons not in a storm shelter or tornado safe room, which are scarce in the Southeast and other parts of the country.[49] Particularly mobile home park residents may benefit from additional storm shelters. The Federal Tornado Shelters Act provides a basis for grant funds in tornado-prone areas,[50] and some state programs are available to provide financial support to residents who build storm shelters or safe rooms.[51] This study also suggests an important role of media warning systems and tornado sirens in preventing injury. In particular, internet and TV warnings seemed to add protection during this tornado event. Finally, this study indicates a need for mental health services following large tornado emergencies. These services are needed over an extended time period to treat disaster-related traumatization and psychiatric disorders, which are often closely linked to loss events experienced during the emergency.
