# Correction: Injuries and Post-Traumatic Stress following Historic Tornados: Alabama, April 2011

**DOI:** 10.1371/annotation/b9d019c7-2854-4d7a-b2b8-614b9f97cfad

**Published:** 2014-01-30

**Authors:** Thomas Niederkrotenthaler, Erin M. Parker, Fernando Ovalle, Rebecca S. Noe, Jeneita Bell, Likang Xu, Melissa A. Morrison, Caitlin E. Mertzlufft, David E. Sugerman

The fourth author's name is incorrect. The correct name is: Rebecca S. Noe. 

The correct Citation is: Niederkrotenthaler T, Parker EM, Ovalle F, Noe RS, Bell J, et al. (2013) Injuries and Post-Traumatic Stress following Historic Tornados: Alabama, April 2011. PLoS ONE 8(12): e83038. doi:10.1371/journal.pone.0083038.

The correct abbreviation of the fourth author's name in the Author Contributions is: RSN.

